# Comparative performance of stool preprocessing methods for Xpert MTB/RIF Ultra: optimizing diagnostic yield in elderly populations

**DOI:** 10.1128/jcm.00901-26

**Published:** 2026-09-30

**Authors:** Hung-Ling Huang, Chou-Jui Lin, Wei-Chang Huang, Chih-Bin Lin, Cheng-Hui Wang, Meng-Hsuan Cheng, Meng-Rui Lee, Chou-Chin Lan, Tzu-Tao Chen, Ching-Sheng Hung, Li-Na Lee, Jann-Yuan Wang, Ming-Chih Yu, Yi-Wen Huang, Chih-Hsin Lee

**Affiliations:** 1 Department of Internal Medicine, Kaohsiung Medical University Hospital 89234, Kaohsiung, Taiwan; 2 Department of Internal Medicine, School of Medicine, College of Medicine, Kaohsiung Medical University 164790, Kaohsiung, Taiwan; 3 Graduate Institute of Medicine, Kaohsiung Medical University 541443, Kaohsiung, Taiwan; 4 Division of Pulmonary and Critical Care Medicine, Department of Internal Medicine, Kaohsiung Medical University Hospital, Kaohsiung Medical University, Kaohsiung, Taiwan; 5 Tao Yuan General Hospital, Ministry of Health and Welfare 63487, Taoyuan, Taiwan; 6 Department of Post-Baccalaureate Medicine, College of Medicine, National Chung Hsing University 729068, Taichung, Taiwan; 7 Division of Pulmonary Immunology & Infectious Diseases, Department of Chest Medicine, Taichung Veterans General Hospital 40293, Taichung, Taiwan; 8 School of Medicine, College of Medicine, Chung Shan Medical University 568609, Taichung, Taiwan; 9 Division of Chest Medicine, Department of Internal Medicine, Tzu Chi General Hospital, Tzu Chi University 59216, Hualien, Taiwan; 10 School of Medicine, Tzu Chi University 111128, Hualien, Taiwan; 11 Department of Laboratory Medicine, Wan Fang Hospital, Taipei Medical University 38032, Taipei, Taiwan; 12 School of Medical Laboratory Science and Biotechnology, College of Medical Science and Technology, Taipei Medical University 535192, Taipei, Taiwan; 13 Department of Respiratory Therapy, College of Medicine, Kaohsiung Medical University, Kaohsiung, Taiwan; 14 Department of Internal Medicine, National Taiwan University Hospital 568619, Taipei, Taiwan; 15 Department of Internal Medicine, National Taiwan University Hospital Hsin-Chu Branch, Hsin-Chu, Taiwan; 16 Division of Pulmonary Medicine, Taipei Tzu Chi Hospital, Buddhist Tzu Chi Medical Foundation 145204, New Taipei, Taiwan; 17 Division of Pulmonary Medicine, Department of Internal Medicine, Shuang Ho Hospital, Taipei Medical University 38032, New Taipei, Taiwan; 18 Division of Pulmonary Medicine, Department of Internal Medicine, School of Medicine, College of Medicine, Taipei Medical University 243733, Taipei, Taiwan; 19 TMU Research Center for Thoracic Medicine, Taipei Medical University 38032, Taipei, Taiwan; 20 Graduate Institute of Clinical Medicine, College of Medicine, Taipei Medical University 243733, Taipei, Taiwan; 21 Department of Laboratory Medicine, Fu Jen Catholic University Hospital, Fu Jen Catholic University 34903, New Taipei, Taiwan; 22 Department of Internal Medicine, College of Medicine, National Taiwan University 38005, Taipei City, Taiwan; 23 Department of Internal Medicine, Kaohsiung Municipal Min-Sheng Hospital 38027 , Kaohsiung, Taiwan; 24 Pulmonary Research Center, Wan Fang Hospital, Taipei Medical University 38032, Taipei, Taiwan; 25 Division of Pulmonology, Department of Internal Medicine, Wan Fang Hospital, Taipei Medical University, Taipei, Taiwan; 26 School of Respiratory Therapy, College of Medicine, Taipei Medical University 243733, Taipei, Taiwan; 27 Chang-Hua Hospital, Ministry of Health and Welfare 106137, Puxin Township, Changhua County, Taiwan; 28 Institute of Medicine, Chung Shan Medical University 568610, Taichung, Taiwan; Mayo Clinic Minnesota, Rochester, Minnesota, USA

**Keywords:** stool Xpert Ultra, stool preprocessing methods, elderly, TB diagnosis

## Abstract

Diagnostic delays in elderly patients with pulmonary tuberculosis (PTB) frequently arise from difficulty obtaining adequate sputum specimens, yet evidence supporting stool-based Xpert MTB/RIF Ultra (Xpert Ultra) as a noninvasive alternative in adults remains limited. This prospective multicenter study conducted in Taiwan enrolled 483 participants, including 398 aged ≥65 years, of whom 167 had PTB, and 316 did not. Three stool preprocessing approaches were compared: homogenization and centrifugation (Stool-I), bead beating and chemical lysis (Stool-II), and a centrifuge-free protocol (Stool-III), with diagnostic performance assessed against a composite reference standard (CRS) and respiratory mycobacterial culture. Sputum Xpert Ultra achieved an overall sensitivity of 85.6% and specificity of 98.4%, while respiratory smear and culture were positive in 66.5% and 86.8% of PTB cases, respectively. Among stool preprocessing methods, Stool-I demonstrated the highest sensitivity, significantly outperforming Stool-III (73.1% vs 62.3%; *P* < 0.0001), while all three methods maintained high specificity of ≥98.4%. In subgroup analyses, Stool-I reached 90.7% sensitivity in patients with radiographic cavitation, and 68.6% in those unable to produce sputum. Notably, stool Xpert Ultra identified 29.2% of PTB cases that had initially tested negative on respiratory Xpert Ultra, highlighting its value as a complementary diagnostic tool. In conclusion, these findings indicate that stool-based Xpert Ultra is a highly specific, noninvasive adjunct for PTB diagnosis in elderly and sputum-scarce patients. With optimized preprocessing and context-specific implementation, this approach has the potential to improve diagnostic yield, reduce the need for invasive procedures, and shorten diagnostic delays across diverse healthcare settings.

**IMPORTANCE:**

Tuberculosis (TB) remains a leading infectious cause of death worldwide, and diagnosis in elderly patients is often limited by inadequate sputum production and the need for invasive respiratory sampling. This study demonstrates that stool, a simple and noninvasive specimen, can be used with Xpert MTB/RIF Ultra to reliably detect *Mycobacterium tuberculosis* in this vulnerable population. Among the preprocessing methods evaluated, the centrifugation-based approach achieved the highest diagnostic sensitivity while maintaining excellent specificity. Notably, stool testing identified nearly 30% of tuberculosis cases that were initially negative on respiratory specimen testing, supporting its role as a complementary diagnostic strategy. These findings highlight a practical and patient-friendly approach that may reduce diagnostic delays, limit the need for invasive procedures, and improve TB care in an aging population that is particularly vulnerable to delayed diagnosis and adverse outcomes.

## INTRODUCTION

As a leading cause of preventable death, tuberculosis (TB) accounted for 10.6 million new cases and 1.3 million deaths worldwide in 2023 (1). In Taiwan, the burden remains substantial, with the epidemic increasingly concentrated among older adults. The incidence reaches up to 90.0 per 100,000 in those aged ≥65 years, which is more than three times the national average (2). Older adults have poorer outcomes and higher mortality, as well as longer delays in diagnosis, partly because of atypical symptoms, frailty, and difficulty producing adequate sputum (3–5). Pulmonary tuberculosis (PTB) diagnosis relies on radiography and sputum testing, but adequate sputum is often unobtainable in frail, cognitively impaired, or institutionalized patients (4). Invasive alternatives, such as induced sputum, gastric lavage, or bronchoscopic sampling, are uncomfortable and burdensome for patients and are resource-intensive, limiting their broad implementation. Xpert MTB/RIF and Xpert MTB/RIF Ultra (Xpert Ultra) rapidly detect *Mycobacterium tuberculosis* (MTB) and rifampicin resistance, with approximately 91% sensitivity and 95% specificity in respiratory specimens (6) and 70%–89% sensitivity in extrapulmonary specimens (7).

The WHO recommends the stool-based Xpert Ultra as an initial diagnostic test for children who cannot produce sputum (8). Respiratory specimen collection is challenging in pediatric TB, and culture yields are as low as 10%–15%; stool provides an accessible, noninvasive specimen for molecular detection of MTB DNA originating from swallowed sputum (9). Stool Xpert Ultra has demonstrated 53% sensitivity and 98% specificity against a microbiological reference standard and increased bacteriological confirmation by 38.6% overall (9–11).

A recent meta-analysis estimated that 23% of adults with presumptive TB are unable to provide self-expectorated sputum samples (12), highlighting a substantial limitation of sputum-based diagnosis. This challenge may be even greater in older adults because of frailty, impaired cough, and cognitive dysfunction. Although direct evidence on sputum quality among older adults with presumptive TB is limited, a study of geriatric patients with pneumonia illustrates this difficulty: sputum could be obtained from only 47 of 101 patients aged ≥65 years, and only 18 of these 47 specimens (38%) were of adequate quality for bacteriological evaluation (13).

Evidence on the performance of the stool Xpert Ultra test in adults, particularly older individuals, remains limited. Among adults with bacteriologically confirmed PTB, stool Xpert Ultra sensitivity ranges from 60.4% to 86.6%, with specificity of 94% to 100%, depending on bacillary burden, disease severity, HIV status, preprocessing, and DNA extraction (9, 14–16). Importantly, there is no validated stool preprocessing protocol for older adults, whose stool characteristics, medication exposures, and possibly lower bacterial burden may affect DNA recovery and polymerase chain reaction accuracy (17).

Therefore, we conducted this study to evaluate the diagnostic performance of various stool-processing methods for Xpert Ultra, with particular emphasis on elderly patients and those unable to produce adequate sputum.

## MATERIALS AND METHODS

### Study design and enrolled populations

This prospective multicenter study was conducted across 11 hospitals in Taiwan from January 2023 to December 2024. Institutional review board approval was obtained at all participating sites (details in the Supplemental material), and written informed consent was provided by participants or their legal guardians. This study employed a head-to-head design to compare the diagnostic performance of stool Xpert Ultra across three preprocessing methods with Xpert Ultra performed on respiratory specimens. The primary outcomes were the sensitivity and specificity of Xpert Ultra for diagnosing PTB against a composite reference standard (CRS), with secondary analyses across clinically relevant subgroups.

Eligible participants were patients with suspected PTB who were treatment-naïve or had received anti-TB therapy for less than 2 weeks. Clinically suspected PTB cases were defined as patients presenting with relevant respiratory or systemic symptoms, such as cough, sputum production, hemoptysis, dyspnea, fever, fatigue, or weight loss, or relevant laboratory abnormalities, such as anemia or elevated inflammatory markers (18). Eligibility further requires the presence of either abnormal chest radiography or computed tomography (CT) imaging suggestive of PTB and with at least one of the following: (i) age ≥65 years, or (ii) cognitive impairment, altered consciousness, or physical disabilities that preclude spontaneous sputum collection. Exclusion criteria include the inability to collect a stool sample with adequate consistency and quantity.

### Composite diagnostic standard for PTB

Final classification was based on a CRS incorporating clinical, radiological, conventional microbiological, histopathological, treatment response, and longitudinal follow-up data. Patients were classified as CRS-PTB when the overall evidence supported active PTB and as non-PTB when PTB was excluded. Culture-confirmed PTB required isolation of MTB in respiratory specimens. Patients with exclusively extrapulmonary TB without pulmonary involvement were classified as non-PTB. Cases with diagnostic uncertainty were reviewed by a multidisciplinary team of at least three experienced specialists and were reassessed as culture results, treatment responses, alternative diagnoses, and follow-up information became available. Final classification was reached by consensus after at least 12 months of follow-up. Adjudicators were blinded to stool Xpert Ultra results, which were not included in the CRS. Because adjudicators did not make independent ratings, inter-rater agreement was not assessed. The number of cases requiring multidisciplinary discussion was not prospectively recorded.

### Stool sample collection and preprocessing

Stool specimens of at least 2 g were collected using standardized procedures, either self-collected or with assistance. Solid samples were preferred, and diaper or bedside collections were allowed when required. The collected samples were placed in sterile containers, kept at room temperature, and delivered to the central laboratory at Wan Fang Hospital within 48 hours, where trained personnel performed all preprocessing and Xpert Ultra testing using uniform standard operating procedures.

Three stool preprocessing methods were applied prior to Xpert Ultra testing. Stool-I involved homogenization, sedimentation, chemical decontamination, and centrifugation, following workflows commonly used in reference laboratories to enrich mycobacteria and reduce inhibitors (19). Stool-II employed bead-based lysis, the addition of AL reagent (Qiagen), and high-speed centrifugation to enhance mycobacterial disruption and improve DNA yield through combined chemical and mechanical lysis (20). Stool-III followed the WHO-recommended simple one-step (SOS) protocol, in which stool was mixed with sample reagent (Cepheid, USA), shaken, and allowed to settle by gravity before being loaded into the cartridge (8). The details of the three preprocessing steps were described in the Supplemental material.

### Statistical analysis

Descriptive statistics summarized demographic and clinical data. Categorical variables were expressed as counts and percentages, and continuous variables as mean ± SD or median (IQR). Group differences were analyzed using a *t*-test or a Mann–Whitney *U* test for continuous variables and a chi-square or Fisher’s exact test for categorical variables.

Diagnostic sensitivity and specificity were calculated with respect to the CRS. Differences across specimen types and processing methods were assessed using McNemar’s test. Diagnostic performance was evaluated using an intention-to-test approach. Errors in Xpert Ultra results were retained in the denominator and classified as unsuccessful MTB detections and uninterpretable rifampicin resistance results. Trace results were classified as positive in the primary analysis and as negative in the sensitivity analysis. Two-tailed *P* < 0.05 was considered significant. Analyses were performed using SPSS software.

### Assessment of rifampicin interpretability

Rifampicin interpretability was defined as a definitive Xpert Ultra result of either rifampicin resistance detected or not detected. Results reported as MTB not detected or rifampicin resistance indeterminate were considered uninterpretable. The rifampicin interpretability rate was calculated as the proportion of tests that provided these definitive results among all enrolled patients with CRS-PTB.

### Sample size determination

The sample size was determined *a priori* to compare paired diagnostic sensitivities across stool preprocessing methods and respiratory testing. Detecting a 10% absolute difference in sensitivity, with a two-sided α of 0.05 and 80% power, required 152 patients with CRS-defined PTB. Assuming 10% attrition and a PTB prevalence of 30%–35%, the target enrollment was approximately 535 participants.

## RESULTS

### Clinical characteristics of study participants

A total of 483 participants completed both sputum and stool Xpert Ultra and were included in the final analysis (details of participant flow in the Supplemental material). Among them, 105/483 (21.7%) had cognitive impairment or physical disabilities that precluded spontaneous sputum collection, and 167/483 (34.6%) were diagnosed with CRS-based PTB, including 145/167 (86.8%) who were culture-confirmed (Tables 1 and 2). In comparison with non-PTB patients, those with PTB were significantly younger and less likely to have hypertension, diabetes mellitus, asthma, chronic obstructive pulmonary disease, or coronary artery disease. Cough, malaise, poor appetite, and weight loss were more common in PTB patients, whereas mucus production and dyspnea were more common among non-PTB subjects. Radiographically, cavitation was significantly more prevalent in the PTB group ([54/167, 32.3%] vs [42/316, 13.3%]; *P* < 0.001).

**TABLE 1:** Demographic and clinical characteristics of study participants stratified by disease status^*b*^^,^^*c*^

	Total(*n* = 483)	Non-PTB(*n* = 316)	PTB (*n* = 167)	*P* value
Age, years (mean ± SD)	75.1 ± 15.7	77.4 ± 14.6	70.6 ± 16.8	<0.001
Male sex	342 (70.8%)	217 (68.7%)	125 (74.9%)	0.155
BMI, kg/m^2^ (mean ± SD)	21.4 ± 4.3	21.5 ± 4.3	21.2 ± 4.3	0.451
Comorbidities				
Hypertension	227 (47.0%)	161 (51.0%)	66 (39.5%)	0.017
Diabetes mellitus	140 (29.0%)	102 (32.3%)	38 (22.8%)	0.028
Asthma/COPD	138 (28.6%)	119 (37.7%)	19 (11.4%)	<0.001
CKD, stages 3–5	54 (11.2%)	35 (11.1%)	19 (11.4%)	0.920
End-stage renal disease	6 (1.24%)	4 (1.27%)	2 (1.20%)	>0.999
Coronary artery disease	174 (36.0%)	128 (40.5%)	46 (27.5%)	0.005
Malignancy^*a*^	61 (12.6%)	36 (11.4%)	25 (15.0%)	0.260
Autoimmune and immune-mediated diseases^*a*^	13 (2.7%)	5 (1.6%)	8 (4.8%)	0.07
Liver cirrhosis	9 (1.9%)	4 (1.3%)	5 (3.0%)	0.287
Clinical presentation				
Fever	108 (22.4%)	71 (22.5%)	37 (22.2%)	0.938
Cough	155 (32.1%)	85 (26.9%)	70 (41.9%)	<0.001
Mucus production (sputum)	252 (52.2%)	180 (57.0%)	72 (43.1%)	0.004
Malaise	48 (9.9%)	24 (7.6%)	24 (14.4%)	0.018
Dyspnea	148 (30.6%)	108 (34.2%)	40 (24.0%)	0.020
Poor appetite	62 (12.8%)	25 (7.91%)	37 (22.2%)	<0.001
Weight loss	59 (12.2%)	28 (8.9%)	31 (18.6%)	0.002
Inability to self-report symptoms	22 (4.6%)	13 (4.1%)	9 (5.4%)	0.523
Chest radiographic findings				
Cavitation	96 (19.9%)	42 (13.3%)	54 (32.3%)	<0.001
Pleural effusion	126 (26.1%)	77 (24.4%)	49 (29.3%)	0.236

^*a*^ Details on the distribution of malignancy types and autoimmune or immune-mediated diseases are provided in the Supplemental material.

^*b*^ BMI, body mass index; CKD, chronic kidney disease; COPD, chronic obstructive pulmonary disease; PTB, pulmonary tuberculosis; SD, standard deviation.

^*c*^ Data are presented as mean ± SD for continuous variables or number (%) for categorical variables. *P* values were calculated using Student’s *t*-test, *χ* test, or Fisher’s exact test.

**TABLE 2:** Characteristics and microbiological findings of respiratory specimens, stratified by tuberculosis disease status^*a*^

Characteristic/finding	Total (*n* = 483)	Non-PTB (*n* = 316)	PTB (*n* = 167)	*P* value
Specimen collection setting				
Home	107 (22.2%)	69 (21.8%)	38 (22.8%)	0.812
Long-term care facility	6 (1.2%)	4 (1.3%)	2 (1.2%)	>0.999
Hospital	370 (76.6%)	243 (76.9%)	127 (76.1%)	0.840
Specimen collection method				
Self-expectorated sputum	367 (76.0%)	235 (74.4%)	132 (79.0%)	0.254
Suctioned sputum	105 (21.7%)	77 (24.4%)	28 (16.8%)	0.053
Bronchial washing	11 (2.3%)	4 (1.3%)	7 (4.2%)	0.046
Microbiological results				
Acid-fast smear				
Negative	345 (71.4%)	289 (91.5%)	56 (33.5%)	<0.001
1+ or 2+	103 (21.3%)	23 (7.3%)	80 (47.9%)	<0.001
3+ or 4+	35 (7.2%)	4 (1.3%)	31 (18.6%)	<0.001
Respiratory specimen culture				
MTB	145 (30.0%)	0	145 (86.8%)	<0.001
NTM	62 (12.8%)	60 (19.0%)	2 (1.2%)	<0.001
Xpert Ultra MTB detected in respiratory specimens	148 (30.6%)	5 (1.6%)	143 (85.6%)	<0.001
Trace	16 (3.3%)	3 (1.0%)	13 (7.8%)	<0.001

^*a*^ MTB, *Mycobacterium tuberculosis* complex; NTM, non-tuberculous mycobacteria; PTB, pulmonary tuberculosis.

### Microbiological characteristics of respiratory and stool specimens

As shown in Table 2, most specimens were collected in a hospital (370/483, 76.6%). Self-expectorated sputum was the predominant type of respiratory specimens in both groups (132/167, 79.0% and 235/316, 74.4% in the PTB and non-TB groups, respectively), whereas bronchial washing was more frequent in the PTB group (4.2% vs 1.3%, *P* = 0.046). Among the 167 PTB patients, respiratory specimen-based Xpert Ultra and MTB culture were positive in 85.6% and 86.8%, respectively.

The stool characteristics assessed using the Bristol Stool Scale (21) did not differ significantly between groups, with type 6 being the most common. Sensitivity of stool Xpert Ultra in the CRS-PTB reached 73.1%, 68.9%, and 62.3% for Stool-I, Stool-II, and Stool-III, respectively. The numbers of “trace” MTB detections were 20, 17, and 23 for Stool-I, Stool-II, and Stool-III, respectively, compared with 16 for respiratory specimens. Stool Xpert Ultra positivity in the non-PTB group was low (0.6%, 1.6%, and 0%, respectively), indicating high specificity across all stool preprocessing methods. Reclassifying trace results as negative reduced sensitivity by 7.8–13.8 percentage points, while specificity improved by no more than 1.0 percentage point (Table S3). Among the 431 patients whose stool culture were performed, MTB was isolated in 12.4% (18/145) patients in the PTB group and in none (0/286) of the non-PTB patients. Among the 18 PTB patients isolating MTB in stool culture, Xpert Ultra was positive in 17, 17, and 17 using Stool-I, Stool-II, and Stool-III, respectively. Among 127 PTB cases whose stool culture did not yield MTB, Xpert Ultra was positive in 89, 83, and 73 using Stool-I, Stool-II, and Stool-III, respectively.

### Diagnostic performance of Xpert Ultra for respiratory specimens and stool with different preprocessing methods

**Fig 1:**
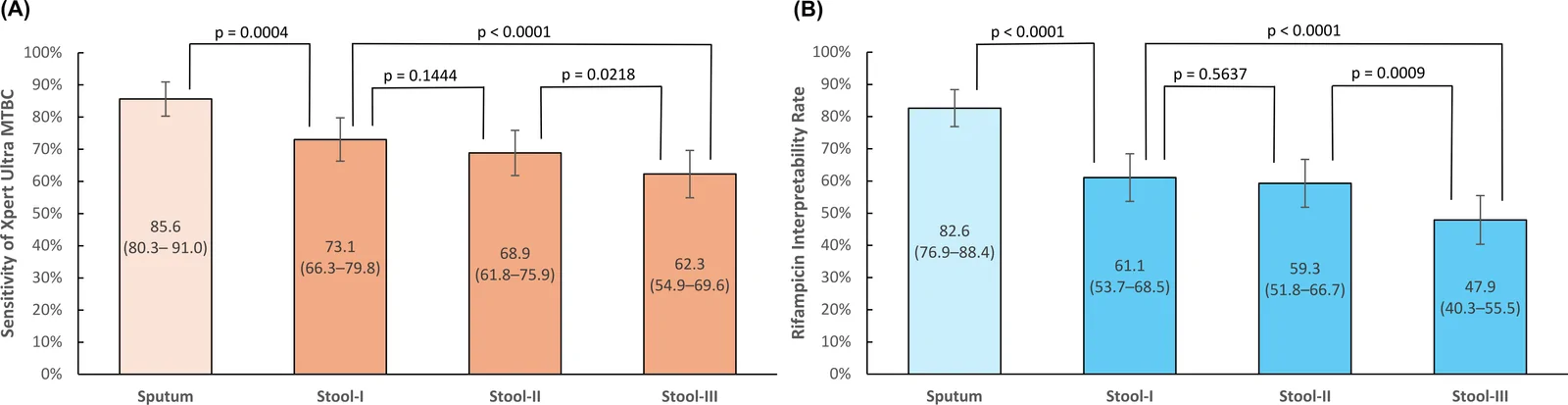
The diagnostic performance of Xpert Ultra for the detection of (**A**) *Mycobacterium tuberculosis* complex (MTBC) and (**B**) rifampicin (RIF) resistance across different specimen types and stool preprocessing methods.

Figure 1 summarizes the sensitivities and rifampicin interpretability rates of Xpert Ultra across specimen types and stool preprocessing methods. For MTB detection, sputum Xpert Ultra demonstrated the highest sensitivity (85.6%, 95% CI: 75.9%–90.1%) with a specificity of 98.4% (95% CI: 97.0%–99.8%). Among the three stool-based protocols, Stool-I achieved the greatest sensitivity (73.1%, 95% CI: 65.9%–79.2%), followed by Stool-II (68.9%, 95% CI: 61.5%-75.4%) and Stool-III (62.3%, 95% CI: 54.7%–69.3%). All three methods demonstrated high specificity (98.4%–100%) for MTB detection (Table 3).

**TABLE 3:** Characteristics and microbiological findings of stool specimens, stratified by tuberculosis disease status^*a*^

	Sensitivity (95% CI) vs CRS-PTB	Specificity (95% CI) vs CRS-PTB	Sensitivity (95% CI) in culture-confirmed PTB	Valid results *n*/*N* (%)	Trace results *n*/*N* (%)	RIF interpretability rate (95% CI)
Stool-I	73.1 (65.9–79.2)	99.4 (98.5–100.0)	79.3 (72.0–85.1)	471/483 (97.5%)	20/483 (4.1%)	61.1 (53.7–68.5)
Stool-II	68.9 (61.5–75.4)	98.4 (97.0–99.8)	75.9 (68.3–82.1)	479/483 (99.2%)	17/483 (3.5%)	59.3 (51.8–66.7)
Stool-III	62.3 (54.7–69.3)	100.0 (100.0–100.0)	69.7 (61.7–76.6)	480/483 (99.4%)	23/483 (4.8%)	47.9 (40.3–55.5)

^*a*^ CI, confidence interval; CRS, composite-reference standard; PTB, pulmonary tuberculosis.

Among non-PTB patients, Xpert Ultra detected MTB in sputum, Stool-I, and Stool-II from five, two, and five patients, respectively. Of the 10 patients with at least one positive result, respiratory cultures were negative in seven and yielded NTM in three. Except for one patient with TB pericarditis without pulmonary involvement, none received anti-TB treatment. None developed evidence of active PTB during more than 12 months of follow-up (details in the Supplemental material).

Compared to sputum, sensitivity of stool-I was lower (*P* < 0.001, Fig. 1A) (*P* < 0.001). Significant differences were also noted between Stool-I and Stool-III (*P* < 0.001) and between Stool-II and Stool-III (*P* = 0.022).

Among CRS-PTB cases, the rifampicin interpretability rate of sputum specimens was 82.6% (95% CI: 76.9%–88.4%) (Fig. 1B), which was higher than those in stool specimens, with Stool-I at 61.1% (95% CI: 53.7%–68.5%), Stool-II at 59.3% (95% CI: 51.8%–66.7%), and Stool-III at 47.9% (95% CI: 40.3%–55.5%).

**Fig 2:**
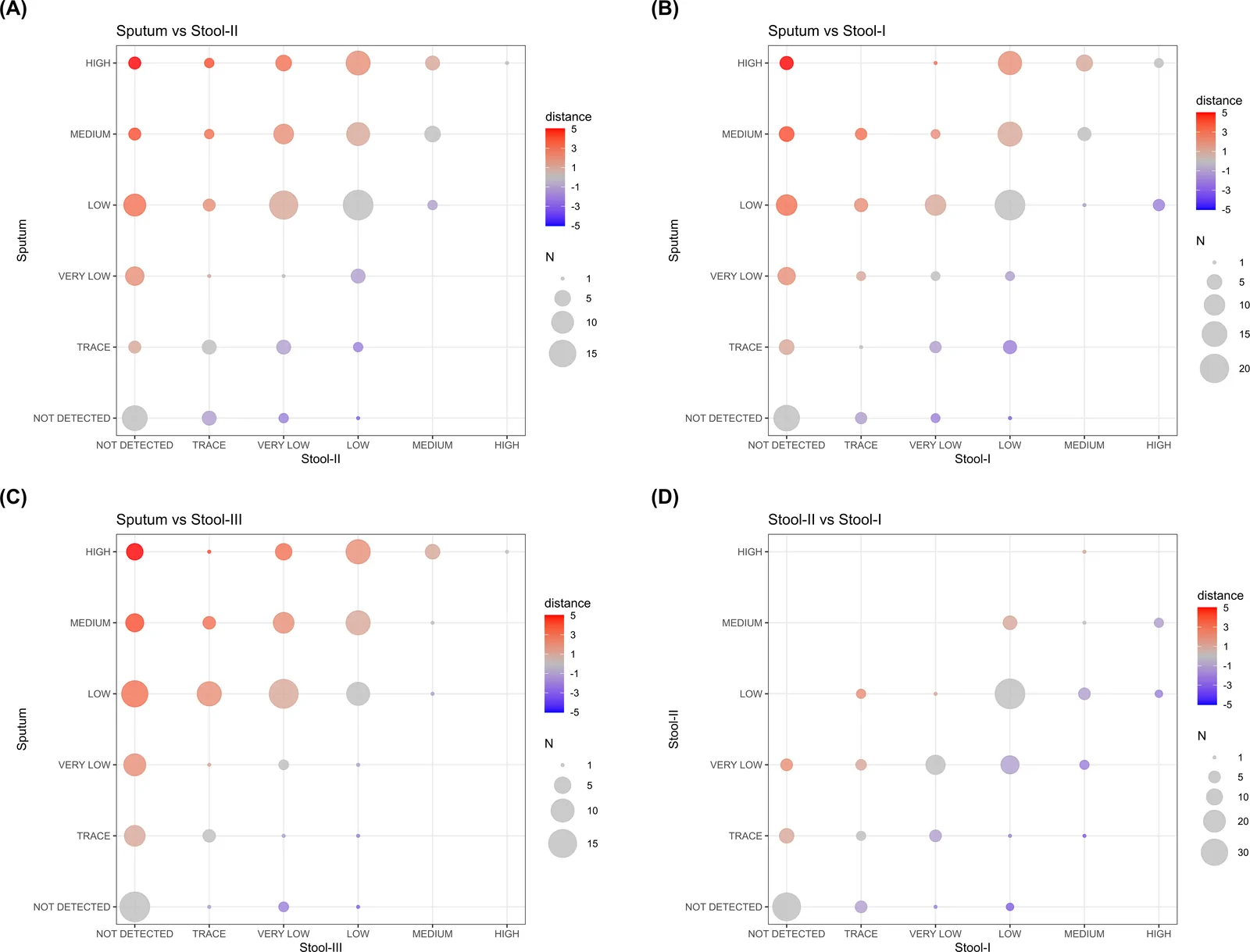
Concordance analysis of semi-quantitative PCR results for *Mycobacterium tuberculosis* detection in sputum versus stool samples across different processing methods. Bubble plots illustrate the pairwise comparisons of semi-quantitative results (categories: not detected, trace, very low, low, medium, high) among sputum samples and stool samples preprocessed using different protocols: (**A**) sputum versus Stool-II; (**B**) sputum versus Stool-I; (**C**) sputum versus Stool-III; and (**D**) Stool-II versus Stool-I. In each plot, the bubble size (*N*) is proportional to the frequency of sample pairs falling within a specific intersection of categories. The color gradient denotes the semi-quantitative difference between the paired results: gray indicates perfect concordance (diagonal), while red and blue intensities indicate the magnitude and direction of the discrepancy (rank difference) between the two methods. Overall, sputum samples yielded higher semi-quantitative results than stool samples preprocessed by any of the three methods. While Stool-I and II performed comparably overall, Stool-II exhibited slightly higher semi-quantitative intensities in samples with low bacterial loads.

Figure 2 presents the concordance of semi-quantitative Xpert Ultra results between sputum and stool specimens across the three processing methods. The bacterial burden categories showed a consistent ordinal trend across specimen types: higher bacillary loads in sputum tended to be accompanied by higher bacillary loads in stool, whereas lower sputum loads corresponded to lower-level or negative stool results. Sputum generally exhibited higher semi-quantitative detection levels than their corresponding stool specimens.

### Subgroup analysis of Xpert Ultra diagnostic performance in sputum and stool specimens with different preprocessing methods

**Fig 3:**
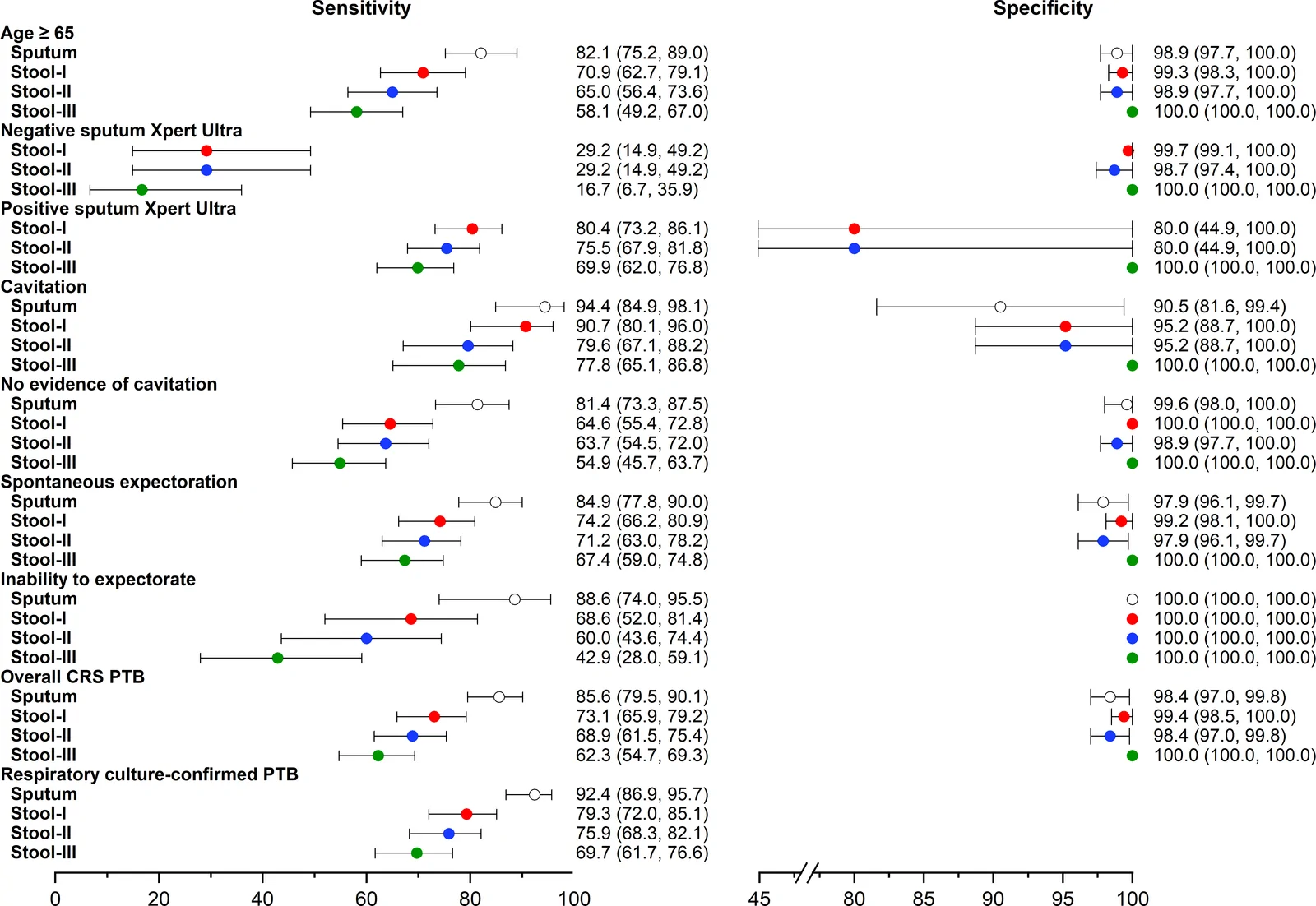
Subgroup analyses of Xpert Ultra diagnostic performance across specimen types and stool preprocessing methods. Forest plots show the sensitivity (left panel) and specificity (right panel) of Xpert Ultra testing using respiratory specimens and stool specimens processed with Stool-I, Stool-II, or Stool-III. Diagnostic performance was evaluated within clinically relevant subpopulations defined by age, respiratory Xpert Ultra result, cavitation, and ability to expectorate sputum, as well as in the overall composite reference standard (CRS)-defined population and among patients with respiratory culture-confirmed PTB. Point estimates are presented with corresponding 95% confidence intervals (CI). Open circles indicate respiratory specimens; red, blue, and green circles indicate Stool-I, Stool-II, and Stool-III, respectively. For each subgroup, sensitivity was calculated among patients classified as pulmonary tuberculosis (PTB) by the CRS, whereas specificity was calculated among CRS-classified non-PTB patients within the same subgroup. The specificity among the positive sputum Xpert Ultra subgroup involves five CRS-classified non-PTB patients, of whom four tested negative with Stool-I and II and all five tested negative with Stool-III. Consequently, the corresponding specificity estimates were 80% (4/5) for Stool-I and II and 100% (5/5) for Stool-III. Because of this small denominator, these estimates have wide CI and should be interpreted cautiously.

Among the 145 patients with culture-confirmed PTB, Stool-I achieved a sensitivity of 79.3% (95% CI: 72.0%–85.1%), which was slightly higher than that observed for CRS-defined PTB, supporting its diagnostic reliability in microbiologically confirmed disease (Fig. 3). Among elderly participants aged ≥65 years, the sensitivities of both sputum- and stool-based methods were consistent with those observed in the overall cohort. Among the five CRS-defined non-PTB patients with positive sputum Xpert Ultra results, four tested negative with Stool-I and II, corresponding to specificities of 80.0% for each method. All five tested negative with Stool-III.

Stool Xpert Ultra performance varied according to sputum molecular results and radiographic severity. Among the 335 patients with negative sputum Xpert Ultra results, 24 had CRS-defined PTB. Stool-I and Stool-II each detected 7/24 (29.2%, 95% CI: 14.9%–49.2%) additional cases and increased overall sensitivity from 85.6% (143/167) to 89.8% (150/167). Stool-III detected 4/24 (16.7%, 95% CI: 6.7%–35.9%), and increased sensitivity to 88.0% (147/167). The NNT to identify one additional PTB case was 47.9 for Stool-I and Stool-II and 83.8 for Stool-III.

Diagnostic sensitivity was higher in patients with cavitary disease than in those without cavitation for both sputum Xpert Ultra (94.4% vs 81.4%) and Stool-I (90.7% vs 64.6%). The sensitivity gap between sputum and Stool-I was substantially greater in non-cavitary disease.

Among the 116 patients unable to expectorate sputum, the noninvasive stool-based Xpert Ultra demonstrated encouraging performance, with sensitivities of 68.6% (95% CI: 52.0%–81.4%), 60.0% (95% CI: 43.6%–74.4%), and 42.9% (95% CI: 28.0%–59.1%) for Stool-I, Stool-II, and Stool-III, respectively. The corresponding NNTs to identify one PTB case were 4.8, 5.5, and 7.7, suggesting that stool-based testing may offer a clinically valuable alternative while avoiding the procedural invasiveness of suction or bronchoscopy.

## DISCUSSION

Stool-I provided the best stool-based performance (73.1% sensitivity and 99.4% specificity) and detected 29.2% of PTB cases missed by sputum Xpert Ultra, supporting its use as a noninvasive add-on test in this predominantly elderly cohort, including patients unable to expectorate sputum. Regarding baseline characteristics, the lower prevalence of diabetes in the PTB group likely reflects their younger age and comparison with a symptomatic non-PTB cohort enriched for comorbidities.

A major strength of this study is the head-to-head comparison of stool preprocessing methods using both CRS-defined and culture-confirmed PTB. Preprocessing substantially influenced diagnostic sensitivity and operational performance in this predominantly elderly cohort, whose complex stool matrix and potentially lower bacillary burden may impair target MTB recovery (22). The homogenization and chemical decontamination used in Stool-I and the combined chemical and mechanical disruption of the mycobacterial cell wall in Stool-II achieved sensitivities of 73.1% and 68.9%, respectively, compared with 62.3% for the centrifugation-free Stool-III. The additional preprocessing steps used in Stool-I and Stool-II may facilitate mycobacterial DNA release and improve target recovery from the complex fecal matrix, whereas the procedural simplicity of Stool-III may facilitate point-of-care implementation, albeit with somewhat lower sensitivity despite its 100% specificity. Stool Xpert Ultra was substantially more sensitive than stool culture, which detected MTB in only 12.4% of PTB cases despite its high specificity. Combined with a shorter turnaround time, this higher diagnostic yield may enable earlier and more effective bacteriological confirmation of PTB.

The observed sensitivity should be interpreted in the context of the reference standard. A CRS includes culture-negative, often paucibacillary cases in which stool Xpert Ultra is less sensitive. Consequently, the overall sensitivity against a CRS depends partly on the proportion of culture-negative cases included and is generally lower than that observed in analyses restricted to culture-confirmed disease. In pediatric studies, stool Xpert Ultra sensitivity was approximately 55%–77% among culture-confirmed cases but decreased to 13%–17% against a CRS (23). In adult studies, the pooled sensitivity among culture-confirmed cases was 73% (9). Similarly, Stool-I achieved a sensitivity of 73.1% against the CRS and 79.3% among respiratory culture-confirmed cases in our study, indicating performance comparable to previous reports.

Stool Xpert Ultra performance varied with disease severity and sputum availability. Sensitivity with Stool-I was higher in cavitary than non-cavitary PTB (90.7% vs 64.6%), consistent with greater ingestion of bacillus-rich respiratory secretions in advanced disease (23). Among sputum-scarce patients unable to expectorate, Stool-I achieved 68.6% sensitivity, supporting its role as a noninvasive rule-in alternative despite lower sensitivity than invasive respiratory sampling (88.6%). In patients with negative sputum Xpert Ultra results, incorporating Stool-I or Stool-II into the diagnostic workflow identified an additional 29.2% of PTB cases. These findings support stool Xpert Ultra as an add-on rather than replacement strategy, particularly for sputum-scarce patients. This sequential strategy may streamline triage, expedite isolation and treatment, and reduce reliance on invasive respiratory sampling in frail older adults. Further comparative studies across healthcare settings are needed to define optimal stool processing within adult TB diagnostic algorithms.

The interpretability rate of rifampicin resistance detection from stool was lower than that from sputum, likely owing to the higher frequency of “Trace,” “Negative,” and “Error” results in the fecal matrix that yield insufficient genomic information. These results emphasize the importance of cautious interpretation of stool-based resistance findings and the need for further refinement of preprocessing protocols to enhance molecular yield.

Our study has several limitations. First, individual adjudicators’ assessments were not prospectively recorded; therefore, inter-rater agreement could not be evaluated. Nevertheless, consensus was reached for all cases, culture-negative PTB constituted only a limited proportion of the cohort, and all non-PTB patients remained free of evidence of active PTB during more than 12 months of follow-up. Second, in patients unable to expectorate, respiratory specimens were obtained by suction or bronchoscopy, which may have enhanced the performance of respiratory Xpert Ultra and widened its apparent sensitivity difference from stool testing. Finally, the small sample sizes of some subgroups limited further stratified analyses.

### Conclusion

Stool Xpert Ultra is a highly specific, noninvasive adjunct for diagnosing PTB in adults, with particular utility in the elderly and those unable to produce adequate sputum. It offers a practical rule-in strategy that may shorten diagnostic delay and reduce transmission risk. Its diagnostic performance is strongly influenced by preprocessing, underscoring the need to tailor implementation to local laboratory capacity and resources, from centrifuge-free protocols in point-of-care settings to more intensive methods in well-equipped laboratories.

## Data Availability

De-identified individual participant data underlying the results reported in this article are available at: Stool Xpert Ultra Open Data.xlsx.

## SUPPLEMENTAL MATERIAL

Supplemental materials jcm.00901-26-s0001.docx Supplemental materials and results, and Tables S1 to S3.

ASM does not own the copyrights to Supplemental Material that may be linked to, or accessed through, an article. The authors have granted ASM a non-exclusive, world-wide license to publish the Supplemental Material files. Please contact the corresponding author directly for reuse.
