# Supplementary material for: Comparative performance of stool preprocessing methods for Xpert MTB/RIF Ultra: optimizing diagnostic yield in elderly populations
Source: J Clin Microbiol. 2026 Sep 30;64(11):e00901-26. doi: 10.1128/jcm.00901-26 (PMC13753799; doi:10.1128/jcm.00901-26)
Supplement: Supplementary file 1 [file jcm.00901-26-s0001.docx]

**Comparative performance of** **stool preprocessing methods for Xpert MTB/RIF Ultra: Optimizing diagnostic yield in elderly populations**

**Hung-Ling Huang ^1,2,3,4^, Chou-Jui Lin ^5^, Wei-Chang Huang ^6,7,8^, Chih-Bin Lin ^9.10^, Cheng-Hui Wang ^11, 12^, Meng-Hsuan Cheng ^1,2,3,13^, Meng-Rui Lee ^14,15^, Chou-Chin Lan ^16,17^, Tzu-Tao Chen ^18,19,20,21^, Ching-Sheng Hung ^11,12^, Li-Na Lee ^22^, Jann-Yuan Wang ^14,23,24^, Ming-Chih Yu ^25,26,27^, Yi-Wen Huang ^28,29^, Chih-Hsin Lee ^19,20,25,26^**

**Affiliations**

^1^ Division of Pulmonary and Critical Care Medicine, ^2^ Department of Internal Medicine, Kaohsiung Medical University Hospital, Kaohsiung City, Taiwan

^3^ Department of Internal Medicine, School of Medicine, College of Medicine, ^4^ Graduate Institute of Medicine, Kaohsiung Medical University, Kaohsiung City, Taiwan

^5^ Tao-Yuan General Hospital, Ministry of Health and Welfare, 1492, Zhongshan Road, Taoyuan District, Taoyuan City, 330, Taiwan

^6^ Department of Post-Baccalaureate Medicine, College of Medicine, National Chung Hsing University, Taichung, 402202, Taiwan

^7^ Division of Pulmonary Immunology & Infectious Diseases, Department of Chest Medicine, Taichung Veterans General Hospital, Taichung, 407219, Taiwan

^8^ School of Medicine, College of Medicine, Chung Shan Medical University, Taichung, 40201, Taiwan

^9^ Division of Chest Medicine, Department of Internal Medicine, Tzu Chi General Hospital, Tzu Chi University, 707 Chung-Yang Road, Section 3, Hualien, 970, Taiwan

^10^ School of Medicine, Tzu Chi University, 701 Zhongyang Road, Section 3, Hualien, 970, Taiwan

^11^ Department of Laboratory Medicine, Wan Fang Hospital, Taipei Medical University, Taipei, Taiwan

^12^ School of Medical Laboratory Science and Biotechnology, College of Medical Science and Technology, Taipei Medical University, Taipei, Taiwan

^13^ Departments of Respiratory Therapy, Kaohsiung Medical University Hospital, Kaohsiung, Taiwan

^14^ Department of Internal Medicine, National Taiwan University Hospital, Taipei, Taiwan

^15^ Department of Internal Medicine, National Taiwan University Hospital Hsin-Chu Branch, Hsin-Chu, Taiwan

^16^ Division of Pulmonary Medicine, Taipei Tzu Chi Hospital, Buddhist Tzu Chi Medical Foundation, New Taipei City, Taiwan

^17^ School of Medicine, Tzu Chi University, Hualien, Taiwan

^18^ Division of Pulmonary Medicine, Department of Internal Medicine, Shuang Ho Hospital, Taipei Medical University, New Taipei City 23561, Taiwan

^19^ Division of Pulmonary Medicine, Department of Internal Medicine, School of Medicine, College of Medicine, Taipei Medical University, Taipei 11031, Taiwan

^20^ TMU Research Center for Thoracic Medicine, Taipei Medical University, Taipei 11031, Taiwan

^21^ Graduate Institute of Clinical Medicine, College of Medicine, Taipei Medical University, Taipei 11031, Taiwan

^22^ Department of Laboratory Medicine, Fu Jen Catholic University Hospital, Fu Jen Catholic University, New Taipei City 24352, Taiwan

^23^ Department of Internal Medicine, College of Medicine, National Taiwan University, Taipei, Taiwan

^24^ Department of Internal Medicine, Kaohsiung Municipal Min-Sheng Hospital, Kaohsiung, Taiwan

^25^ Pulmonary Research Center, Wan Fang Hospital, Taipei Medical University, Taipei, Taiwan

^26^ Division of Pulmonology, Department of Internal Medicine, Wan Fang Hospital, Taipei, Taiwan

^27^ School of Respiratory Therapy, College of Medicine, Taipei Medical University, Taipei, Taiwan

^28^ Chang-Hua Hospital, Ministry of Health and Welfare, 80 Zhongzheng Road, Section 2, Puxin Township, Changhua County, 513, Taiwan

^29^ Institute of Medicine, Chung Shan Medical University, 110 Jianguo North Road, Section 1, Taichung City, 402, Taiwan

**Corresponding author**

**Dr. Chih-Hsin Lee**

**Email:** [**chleetw@tmu.edu.tw**](mailto:chleetw@tmu.edu.tw)

**Tel: +886-2-29307930**

**Fax: +886-2-29302448**

**Methods**

**Study design and enrolled populations**

This prospective, multicenter study was conducted during January 1, 2023, to December 31, 2024 across eleven hospitals in Taiwan, including Taipei Medical University Wan Fang Hospital, Hualien Tzu Chi Hospital, Taichung Veterans General Hospital, Kaohsiung Medical University Chung-Ho Memorial Hospital, National Taiwan University Hospital, National Taiwan University Hospital Hsinchu Branch, Taoyuan General Hospital, Ministry of Health and Welfare, Changhua Hospital, Ministry of Health and Welfare, Fu Jen Catholic University Hospital, Taipei Tzu Chi Hospital. The study was approved by the institutional review boards of all participating hospitals (KMUHIRB-SV(II)-20230022, N202210039, 12-M-097, 112-071-B, SE23217C, 202304008RINC, TYGH112047)

**Clinical data collection**

Demographic and clinical data were recorded at enrollment, including basic characteristics, symptoms, functional status, comorbidities, and radiological severity, with documentation of cavitary disease. Results from routine sputum examinations, including smear microscopy, culture, nucleic acid amplification, and drug susceptibility testing, were collected.

**Stool preprocessing details**
**Stool I:** Stool specimens were processed using an NALC-NaOH-based decontamination and concentration method prior to Xpert MTB/RIF Ultra testing (Cepheid, Sunnyvale, CA, USA). This protocol is similar to reference laboratory workflows designed to enrich bacteria and minimize inhibitors **(1)**. Briefly, 2 mL of stool specimen was placed into a 50 mL centrifuge tube and homogenized with 10 mL of phosphate buffer (0.067 M, pH 6.8; PBS Buffer, Creative Life Science, New Taipei, Taiwan; Cat. No. CMP0400650) by manual shaking at moderate speed. The homogenate was allowed to stand for 15 minutes to permit sedimentation of particulate matter, after which the supernatant was aspirated using a sterile pipette and transferred to a new 50 mL tube.

An equal volume (10 mL) of freshly prepared 3% N-acetyl-L-cysteine (NALC)-NaOH digestion solution (NALC-NaOH; Creative Life Science, Cat. No. CMP0401260), consisting of Digest Solution with 3% NaOH (250 mL) supplemented with 1.25 g of N-acetyl-L-cysteine, was added to the supernatant and mixed thoroughly. The mixture was intermittently agitated on a vortex mixer for approximately 15–20 seconds. Vigorous or prolonged vortexing for more than 30 seconds was avoided to prevent oxidative inactivation of NALC. The specimen was then incubated at room temperature for 15 minutes to allow decontamination.

Following decontamination, sterile phosphate buffer was added to bring the total volume to 40 mL. The tube was capped tightly and mixed by gentle inversion. Tubes were loaded into a centrifuge safety carrier within a biosafety cabinet (BSC), sealed, and then transferred to the centrifuge. Specimens were centrifuged at 3,000 × g for 15 minutes at a temperature below 20°C.

The supernatant was carefully decanted into a waste container. The sediment was resuspended in 3 mL of sterile phosphate buffer and centrifuged again at 3,000 × g for 5 minutes at a temperature below 20°C. Finally, 2 mL of the resulting supernatant was aspirated with a sterile pipette and transferred into the Xpert MTB/RIF Ultra cartridge, taking care to avoid aspirating fecal particulates that could clog the cartridge and cause assay failure. The cartridge was then loaded onto the GeneXpert instrument for testing according to the manufacturer's instructions.

**Stool II:** Stool specimens were processed using a bead-based lysis and homogenization protocol prior to testing with the Xpert MTB/RIF Ultra assay to maximize mycobacterial lysis and DNA yield **(2)**. As a preparatory step, 1 g of polyvinylpyrrolidone (PVP; Millipore, Merck, Cat. No. 5295) and 1 g of glass lysis beads (QIAGEN, Cat. No. 13118-400) were individually weighed using a microbalance and combined in a 50 mL tube, which was stored at room temperature until use.

For each specimen, 0.6-0.8 g of stool was placed into a 50 mL tube containing 2 mL of phosphate buffer (0.067 M, pH 6.8; PBS Buffer, Creative Life Science, New Taipei, Taiwan; Cat. No. CMP0400650) and homogenized by manual shaking at moderate speed. Subsequently, 10 mL of AL lysis buffer (QIAGEN, Cat. No. 19075) was added, and the mixture was again homogenized by shaking. The pre-weighed PVP and glass bead mixture was then added to the tube, followed by further homogenization by shaking.

The specimen was placed on a rotating mixer and continuously inverted at room temperature for 30 minutes to ensure thorough lysis and homogenization. Following mixing, the supernatant was aspirated using a sterile pipette and distributed into three 1.5 mL Eppendorf tubes at 1 mL per tube, taking care to avoid aspirating the sediment at the bottom of the tube.

The three Eppendorf tubes were then centrifuged at high speed (9,500 rpm; approximately 8,275 × g) for 5 minutes. A total of 2 mL of the resulting supernatant was aspirated with a sterile pipette and transferred into the Xpert MTB/RIF Ultra cartridge, taking care to avoid aspirating fecal particulates that could clog the cartridge and cause assay failure. The cartridge was subsequently loaded onto the GeneXpert instrument for testing in accordance with the manufacturer's instructions.

**Stool III:** The centrifuge-free protocol follows the Simple One-Step (SOS) method, as recommended by the World Health Organization for direct testing of stool with the Xpert MTB/RIF Ultra assay **(3)**. Prior to processing, stool consistency was assessed and documented according to the Bristol Stool Scale (4), with specimens classified as Panel A (Bristol types 1–5, solid stool) or Panel B (Bristol types 6–7, liquid stool).

For solid stool specimens (Bristol types 1–5), 0.8 g of stool was weighed and added to 8 mL of Sample Reagent (Cepheid); specimens corresponding to Bristol types 1–2 were manually fragmented into smaller pieces prior to weighing to facilitate homogenization. For liquid stool specimens (Bristol types 6–7), 2 mL of stool was combined with 6 mL of Sample Reagent. The specimen container was tightly capped and shaken vigorously by hand in a vertical motion for 30 seconds; vortexing was avoided to prevent excessive aerosolization and specimen degradation. The mixture was then allowed to stand at room temperature for 10 minutes to permit initial sedimentation of fecal particulates.

This shaking-and-settling cycle was repeated once: the specimen was shaken vertically for 30 seconds, then left to stand at room temperature for an additional 10 minutes, allowing further sedimentation of fecal debris and clarification of the supernatant. Following this second incubation period, 2 mL of the clarified supernatant was aspirated using a sterile disposable pipette, taking care to avoid aspirating fecal particulates. To minimize resuspension of settled debris, the specimen container was kept stationary throughout the aspiration process. The aspirated supernatant was transferred directly into the Xpert MTB/RIF Ultra cartridge, the cartridge lid was closed, and the cartridge was loaded onto the GeneXpert instrument for testing according to the manufacturer's instructions.

**Results**

**Details of enrollment and exclusion**

The study initially enrolled 535 participants who are clinically suspected of pulmonary tuberculosis. Six participants withdrew consent; nine had received anti-tuberculosis treatment for more than two weeks before submitting stool specimens; thirteen did not undergo sputum Xpert Ultra testing; and twenty-four did not return stool specimens. Consequently, 483 participants completed both sputum and stool testing and were included in the final analysis.

**Details of comorbidities:**

**Malignancy:** The total number of cancer cases comprises lung cancer (n=14), colorectal cancer (n=10), prostate cancer (n=9), breast cancer (n=5), bladder cancer (n=5), esophageal cancer (n=3), gastric cancer (n=2), buccal cancer (n=2), ampulla of Vater cancer (n=2), kidney cancer (n=2), thyroid cancer (n=2), pancreatic cancer (n=1), hepatoma (n=1), osteosarcoma (n=1), B-cell lymphoma (n=1), and cervical cancer (n=1).

**Autoimmune and immune-mediated disease:** The total number of cases comprises rheumatoid arthritis (n=6), Sjögren's syndrome (n=2), psoriatic arthritis (n=2), ankylosing spondylitis (n=1), dermatomyositis (n=1), and myasthenia gravis (n=1).

**Table S1. Details of 10 non-PTB cases with positive sputum or stool Xpert Ultra**

| # | Xpert Ultra | | | |  | Culture | | Cavity | Note |
| --- | --- | --- | --- | --- | --- | --- | --- | --- | --- |
|  | **Sputum** | **Stool I** | **Stool II** | **Stool III** |  | **Sputum** | **Stool** |  |  |
| 1 | **Very low** | N | N | N |  | **NTM** | N | No | **TB pericarditis** |
| 2 | **Trace** | N | N | N |  | N | N | **Yes** | **Old TB** |
| 3 | **Trace** | N | N | N |  | N | N | **Yes** | **Old TB** |
| 4 | **Trace** | N | N | N |  | **NTM** | N | **Yes** | **Old TB** |
| 5 | **Low** | **Trace** | **Very low** | N |  | N | N | **Yes** | **Old TB** |
| 6 | N | **Trace** | N | N |  | N | N | **Yes** |  |
| 7 | N | N | **Very low** | N |  | N | N | No |  |
| 8 | N | N | **Trace** | N |  | N | Not done | **Yes** |  |
| 9 | N | N | **Trace** | N |  | N | N | No |  |
| 10 | N | N | **Low** | N |  | **NTM** | N | No |  |

N: negative; NTM: non-tuberculous mycobacteria.

**Table S2.** Characteristics and culture results of stool specimens, stratified by tuberculosis disease status

| **Characteristic / Finding** | **Total (n=483)** | **Non-PTB (n=316)** | **PTB**  **(n=167)** | ***P* value** |
| --- | --- | --- | --- | --- |
| **Stool Patterns (Bristol Stool Scale)** | | | | |
| Type 1 (Separate hard lumps) | 23 (4.8%) | 18 (5.7%) | 5 (3.0%) | 0.198 |
| Type 2 (Lumpy sausage) | 22 (4.6%) | 14 (4.4%) | 8 (4.8%) | 0.852 |
| Type 3 (Sausage with cracks) | 22 (4.6%) | 15 (4.8%) | 7 (4.2%) | 0.781 |
| Type 4 (Smooth sausage/snake) | 52 (10.8%) | 36 (11.4%) | 16 (9.6%) | 0.548 |
| Type 5 (Soft blobs) | 100 (20.7%) | 70 (22.2%) | 30 (18.0%) | 0.276 |
| Type 6 (Mushy / Fluffy pieces) | 223 (46.2%) | 138 (43.7%) | 85 (50.9%) | 0.130 |
| Type 7 (Watery / Liquid) | 33 (6.8%) | 18 (5.7%) | 15 (9.0%) | 0.168 |
| No record | 8 (1.7%) | 7 (2.2%) | 1 (0.6%) | 0.271 |
| **Culture results (available in 431)** | (n=431) | (n=286) | (n=145) |  |
| MTB | 18 (4.2%) | 0 | 18 (12.4%) | < 0.001 |
| NTM | 10 (2.3%) | 9 (3.1%) | 1 (0.7%) | 0.175 |

PTB: pulmonary tuberculosis; MTB: *Mycobacterium tuberculosis* complex; NTM: non-tuberculous mycobacteria.

**Stool Patterns:** Based on the Bristol Stool Scale (4). Types 1–5 are considered solid/semi-solid; Types 6–7 are considered liquid.

**Table S3.** Diagnostic performance of Xpert Ultra according to the classification of trace results

| **Specimen or method** | **Sensitivity**  **trace as positive** | **Sensitivity**  **trace as negative** | **Specificity**  **trace as positive** | **Specificity**  **trace as negative** |
| --- | --- | --- | --- | --- |
| Sputum | 143/167 (85.6%) | 130/167 (77.8%) | 311/316 (98.4%) | 314/316 (99.4%) |
| Stool-I | 122/167 (73.1%) | 104/167 (62.3%) | 314/316 (99.4%) | 316/316 (100.0%) |
| Stool-II | 115/167 (68.9%) | 100/167 (59.9%) | 311/316 (98.4%) | 313/316 (99.1%) |
| Stool-III | 104/167 (62.3%) | 81/167 (48.5%) | 316/316 (100.0%) | 316/316 (100.0%) |

In the primary analysis, Xpert Ultra trace results were classified as positive. The alternative analysis classified trace results as negative. Sensitivity and specificity were calculated against the composite reference standard, with CRS-PTB (n=167) and CRS-non-PTB (n=316) as the respective denominators.

**References**

1.	Kokuto H, Sasaki Y, Yoshimatsu S, Mizuno K, Yi L, Mitarai S. Detection of Mycobacterium tuberculosis (MTB) in Fecal Specimens From Adults Diagnosed With Pulmonary Tuberculosis Using the Xpert MTB/Rifampicin Test. Open Forum Infect Dis. 2015;2(2):ofv074.

2.	Sun L, Liu Y, Fang M, Chen Y, Zhu Y, Xia C, et al. Use of Xpert MTB/RIF Ultra assay on stool and gastric aspirate samples to diagnose pulmonary tuberculosis in children in a high-tuberculosis-burden but resource-limited area of China. Int J Infect Dis. 2022;114:236–43.

3.	Practical manual of processing stool samples for diagnosis of childhood TB. Geneva: World Health Organization; 2022.

4.	O'Donnell L, Virjee J, Heaton K. Detection of pseudodiarrhoea by simple clinical assessment of intestinal transit rate. BMJ: British Medical Journal. 1990;300(6722):439.
